# Revealing key biomarkers and molecular mechanisms associated with di(2-ethylhexyl) phthalate in skin cancer

**DOI:** 10.3389/fmolb.2026.1841556

**Published:** 2026-05-21

**Authors:** Haidong Zhou, Haiyang Wang, Kangqi Xie, Huade Ma, Runze Wu, Dingpeng Ban, Jiahui Wang, Zhonghua Sun, Jihua Wei, Dapeng Zhou

**Affiliations:** 1 Department of Burn Plastic and Wound Repair Surgery, Key Laboratory of Clinical Cohort Research on Bone and Joint Degenerative Diseases of Guangxi, Affiliated Hospital of Youjiang Medical University for Nationalities, Baise, Guangxi, China; 2 Medical School, Nanjing University, Nanjing, Jiangsu, China

**Keywords:** bioinformatics, biomarker, Di(2-ethylhexyl) phthalate, skin cancers, toxicological mechanisms

## Abstract

**Background:**

Di (2-ethylhexyl) phthalate (DEHP), the most widely used phthalate plasticizer, has been implicated in skin cancer. However, its key targets and multi-pathway mechanisms regulating skin cancer onset and progression remain unclear. Therefore, elucidating DEHP’s molecular mechanisms in skin cancer development is crucial for prevention and intervention strategies.

**Methods:**

This study integrates network toxicology, molecular docking, and experimental validation to systematically elucidate the mechanism by which DEHP induces skin cancer. Specifically, we predicted potential DEHP targets and skin cancer-associated targets (melanoma, squamous cell carcinoma, basal cell carcinoma) using multiple databases. Core targets were identified through CytoNCA topological analysis, MCODE module mining, and CytoHubba multi-algorithm integration. Performed GO/KEGG enrichment analysis using the DAVID database; validated the binding potential between DEHP and core targets via molecular docking with AutoDock Vina; and finally verified the abnormal expression profiles of core targets through TCGA/GTEx transcriptomic data, HPA proteomic data, and RT-qPCR experiments in A-375 (human malignant melanoma cells)/Hacat cells.

**Results:**

Initially, this study identified 11 key DEHP-induced skin cancer targets: CTNNB1, ESR1, HIF1A, IL6, MTOR, MYC, STAT3, AKT1, BCL2, CASP3, and CCND1. These targets exhibited specific regulation across different skin cancer subtypes. Subsequently, a four-tier regulatory network linking “DEHP-core targets-pathways-skin cancer” was constructed. Molecular docking confirmed stable binding conformations between DEHP and all 11 key targets, while enrichment analysis revealed their associations with cellular proliferation, apoptosis, inflammatory responses, and core pathways including Jak-STAT and PI3K-Akt/mTOR. Finally, transcriptomic, proteomic, and *in vitro* cellular experiments validated the significant dysregulation of these targets in skin cancer tissues and tumor cells.

**Conclusion:**

Collectively, this study systematically elucidates the toxicological mechanism by which DEHP promotes skin cancer development through subtype-specific pathways regulated by 11 key targets, clarifying its direct binding patterns with core targets and downstream pathway disruption characteristics. This not only fills a research gap in the molecular mechanisms of DEHP-induced skin carcinogenesis but also provides novel biomarkers for environmental exposure prevention and targeted interventions against skin cancer.

## 1 Introduction

Di (2-ethylhexyl) phthalate (DEHP) is the most widely used plasticizer among phthalates (PAEs), and due to its high plasticizing properties, it is extensively employed in various industrial and consumer products, including food packaging materials, medical devices, children’s toys, and personal care products (Astuto et al., 2023; An et al., 2026). Since DEHP primarily binds to polymer matrices through physical mixing rather than covalent bonds, it readily migrates and leaches during product use, aging, or exposure to environmental factors (e.g., light, temperature fluctuations, organic solvents). Consequently, it is widely distributed in environmental media including air, soil, water, and food (Viljoen et al., 2023; Xian et al., 2025). Humans can continuously accumulate DEHP through multiple exposure pathways including dietary intake, skin contact, respiratory inhalation, and medical exposure, making it a ubiquitous environmental pollutant (Yu et al., 2025; Wang H. et al., 2026). As a prototypical endocrine-disrupting chemical (EDC), DEHP’s toxic effects have been extensively documented. It not only causes multifaceted damage to the reproductive, nervous, and metabolic systems but is also closely associated with the development of various cancers, including ovarian, liver, and breast cancers (Chen et al., 2025; Dai et al., 2025; Wang et al., 2025a; Zhang et al., 2025). In recent years, epidemiological investigations and toxicological experimental evidence suggest a potential association between DEHP exposure and increased skin cancer risk (Benjamin et al., 2017; Zaman et al., 2025). As the body’s largest barrier organ, prolonged direct contact with DEHP-containing products or environmental media may lead to DEHP accumulation in skin tissue. This accumulation can induce abnormal proliferation of skin cells, inhibit apoptosis, and disrupt oxidative stress balance, thereby providing a pathophysiological basis for skin cancer development (Andersen et al., 2018; Hopf et al., 2024; Li et al., 2022; Martínez-Ibarra et al., 2024). However, the specific molecular mechanisms and key functional targets by which DEHP regulates skin cancer initiation and progression remain incompletely elucidated. Systematic and in-depth research is still needed to understand how DEHP synergistically drives skin cancer progression through multiple targets and pathways.

Skin cancer is a group of malignant tumors originating in the skin, primarily classified into two major categories based on pathological type: melanoma and non-melanoma skin cancer (NMSC), and NMSC further includes basal cell carcinoma (BCC) and squamous cell carcinoma (SCC) (Diao et al., 2026; St Denis et al., 2025). Epidemiological data indicate that skin cancer ranks among the most prevalent malignant tumors globally. In 2020, new cases exceeded 1.2 million worldwide, accounting for 6.2% of all new cancer diagnoses (Atalay et al., 2025; Ali et al., 2025). Alarmingly, current treatments for skin cancer exhibit significant limitations, such as pronounced side effects from chemotherapy, susceptibility to drug resistance in targeted therapies, and prohibitively high treatment costs for advanced-stage patients, imposing a heavy economic burden on both patient families and healthcare systems (Cortesi et al., 2026; Janssen et al., 2024). The development of skin cancer results from the combined effects of genetic susceptibility and environmental exposure. Beyond classic UV radiation, exposure to environmental pollutants has been confirmed as a significant risk factor (Benjamin et al., 2017; Qian et al., 2025). As a representative of widely prevalent phthalate compounds in the environment, DEHP can enter the human body through multiple routes, including skin contact and dietary intake, and accumulate in skin tissue. Epidemiological and toxicological evidence has indicated an association between DEHP exposure and an increased risk of skin cancer (Benjamin et al., 2017; Zaman et al., 2025). However, the specific molecular mechanisms by which DEHP promotes the initiation and progression of skin cancer remain unclear.

Based on the key scientific questions outlined above, this study systematically elucidated the toxicological mechanisms by which DEHP exposure promotes skin cancer through the integration of network toxicology, molecular docking, and experimental validation. Specifically, this study first jointly predicted potential DEHP targets and skin cancer-related targets through multiple databases. After intersecting and screening common targets, PPI network topology analysis, module mining, and multi-algorithm integration were employed to identify core regulatory targets, constructing a four-tier regulatory network: “DEHP - Core Targets - Pathways - Skin Cancer.” Subsequently, molecular docking validated the direct binding potential and interaction patterns between DEHP and core targets. Finally, TCGA/GTEx transcriptomic data, HPA proteomic data, and *in vitro* cell experiments validated the abnormal expression characteristics of core targets. The findings identified core targets regulated by DEHP in melanoma, squamous cell carcinoma, and basal cell carcinoma, respectively. They revealed the critical roles of Jak-STAT, HIF-1, PI3K-Akt/mTOR pathways. These findings confirm that DEHP synergistically drives abnormal skin cell proliferation, apoptosis suppression, and inflammatory imbalance by directly binding core targets and disrupting downstream pathways. In summary, this study not only fills a gap in understanding the molecular mechanisms of DEHP-induced skin carcinogenesis but also provides novel biomarkers for environmental exposure prevention and targeted interventions against skin cancer.

## 2 Materials and methods

### 2.1 DEHP toxicity analysis

To investigate the toxicological characteristics of DEHP and its potential association with skin cancer development, this study systematically retrieved relevant research literature from databases including PubMed, Google Scholar, China National Knowledge Infrastructure (CNKI), and ToxNet. The focus was on studies examining the relationship between DEHP exposure and skin cancer occurrence (Benjamin et al., 2017; Zaman et al., 2025).

### 2.2 Screening of potential DEHP targets

The SMILES (CCCCC(CC)COC(=O)C1 = CC = CC = C1C(=O)OCC(CC)CCCC) of DEHP were retrieved from the PubChem database (https://pubchem.ncbi.nlm.nih.gov). Subsequently, this study combined multiple online target prediction tools—STITCH (http://stitch.embl.de/), ChEMBL (https://www.ebi.ac.uk/chembl/), Swiss Target Prediction (http://swisstargetprediction.ch/), and PharmMapper (https://lilab-ecust.cn/pharmmapper/) to predict potential targets of DEHP (Wang H. et al., 2026; Sima et al., 2025; Wang et al., 2026b). Finally, after merging and deduplicating the predicted targets from each tool, the corresponding gene names were standardized and unified using the Uniprot database (https://www.uniprot.org).

### 2.3 Prediction and acquisition of skin cancer-related targets

Skin cancer-related targets were predicted and acquired using the online Online Mendelian Inheritance in Man (OMIM, https://omim.org/) and GeneCards (https://www.genecards.org/) (Zhan and Shi, 2025; Yi-Fan and Jian-Rong, 2025). Search keywords were set to “Melanoma skin cancer,” “Squamous cell cancer,” and “Basal cell cancer” to ensure comprehensive prediction and acquisition of relevant targets (St Denis et al., 2025; Atalay et al., 2025; Wang et al., 2025b). In the GeneCards database, the median score of all targets was used as the screening threshold for “score,” identifying targets scoring above this threshold (Cheng et al., 2025). Finally, targets from different sources were integrated and duplicates removed to construct a skin cancer-related target set.

### 2.4 Intersection analysis of DEHP targets and skin cancer targets

To identify potential targets through which DEHP regulates skin cancer, this study performed an intersection analysis between the DEHP target set and the skin cancer target set obtained above. Additionally, a Venn diagram was generated using the Sangerbox database (http://sangerbox.com/home.html) to visually represent the overlapping region between the two target sets (Shen W. et al., 2022).

### 2.5 Functional enrichment analysis

To clarify the biological processes and signaling pathways involving key targets and thereby reveal the potential molecular mechanisms by which DEHP promotes skin cancer development, this study performed Gene Ontology (GO) and Kyoto Encyclopedia of Genes and Genomes (KEGG) enrichment analyses on the intersecting targets. GO analysis and KEGG analysis were performed using the DAVID database (https://david.ncifcrf.gov/) (Wang H. et al., 2025; Sherman et al., 2022; Shen et al., 2025; Wang et al., 2026c). To clearly present the analysis results, the top 10 enriched GO terms (covering biological processes, molecular functions, and cellular components) and KEGG pathways were visualized using the bioinformatics analysis platform (https://www.bioinformatics.com.cn).

### 2.6 Protein-protein interaction (PPI) network construction and analysis

To identify key genes from the intersecting targets, this study conducted a stepwise protein-protein interaction (PPI) network analysis. First, the intersecting gene list was uploaded to the STRING database (https://string-db.org/) with species restricted to “*Homo sapiens*” to construct a foundational PPI network (Szklarczyk et al., 2019). Subsequently, the obtained PPI network data was imported into Cytoscape software for network visualization and preliminary topological analysis (Shannon et al., 2003). Specifically, the CytoNCA plugin was used to calculate topological parameters such as Betweenness, Closeness, and Degree for network nodes. The median values of each parameter served as the screening criterion to obtain the initial core gene set (Tang et al., 2015; Wei et al., 2025). Subsequently, the MCODE plugin was applied to perform module mining on the core network, identifying and extracting functionally interconnected subnetworks (Guo et al., 2023). Finally, based on the CytoHubba plugin, eight algorithms—Maximum Neighborhood Component (MNC), Degree, Edge Percolated Component (EPC), Closeness, Radiality, Betweenness, Stress, and Maximum Subgraph Centrality (MSS) to screen the top 10 hub genes ranked by each algorithm. Cross-algorithm analysis was then performed to identify core targets of DEHP-promoted skin carcinogenesis (Guan et al., 2025). To further elucidate the biological functions of these core targets, enrichment analysis was conducted.

### 2.7 Construction of the “compound-target-pathway-disease” network

To systematically present the multi-level regulatory relationships among DEHP, core targets, enriched pathways, and skin cancer, this study constructed an associative network model linking “DEHP-Target-Pathway-Skin Cancer.” Based on database-predicted DEHP-target relationships, enrichment pathway information for core targets, and the association between pathways and different skin cancer subtypes, this study constructed an attribute matrix encompassing these four hierarchical relationships, achieving systematic integration and visual representation of the molecular interaction network.

### 2.8 Molecular docking

To validate the interaction potential between core targets and DEHP, molecular docking simulations were conducted (Wei et al., 2025; Yi et al., 2025). First, protein structure files for 11 target proteins—CTNNB1, ESR1, HIF1A, IL6, MTOR, MYC, STAT3, AKT1, BCL2, CASP3, and CCND1. Simultaneously, the structural file for DEHP and octocrylene were obtained from the PubChem database (https://pubchem.ncbi.nlm.nih.gov/). Subsequently, water molecules were removed from the receptor proteins using PyMOL software. DEHP ligands underwent hydrogenation modification and format conversion via AutoDockTools software (Shen et al., 2023). AutoDock Vina was then employed for docking calculations, with the active pockets of each target protein serving as the grid center. Finally, PyMOL software visualized the three-dimensional conformation of the docked complexes, while Discovery Studio 2019 Client analyzed interaction patterns between these complexes.

### 2.9 Differential expression analysis of key proteins in normal vs. tumor tissues

To investigate the expression characteristics of key proteins in skin cancer, this study selected melanoma—a highly malignant and clinically significant tumor—as the analysis subject, focusing on evaluating the expression patterns of 11 core proteins. This study integrated standardized RNA sequencing datasets from TCGA (tumor samples) and GTEx (normal tissue samples) based on the UCSC XENA database (https://xenabrowser.net/datapages/), with data presented in transcripts per million (TPM). TCGA tumor samples for Skin Cutaneous Melanoma (SKCM) were extracted from the pan-cancer dataset and compared with corresponding normal tissue data from GTEx. Differential expression analysis was performed using the Wilcoxon test. At the protein expression level, this study systematically compared the expression of these 11 key proteins in SKCM tumor tissue versus corresponding normal tissue using immunohistochemistry results from the Human Protein Atlas (HPA) database (https://www.proteinatlas.org/) (Shen T. et al., 2022). The HPA is a publicly available database that provides systematic protein expression profiles across human normal tissues, and cancer tissues using antibody-based immunohistochemistry and transcriptomics. All IHC images in the HPA are generated using standardized tissue microarray protocols, stained with DAB-labeled antibodies and counterstained with hematoxylin (Huang et al., 2023; Liu et al., 2022; Ma et al., 2021; Uhlen et al., 2017).

### 2.10 Experimental validation

To validate the results of the bioinformatics analysis and evaluate the effects of DEHP, human malignant melanoma A-375 cells and human immortalized keratinocytes (HaCaT) were cultured in DMEM medium containing 10% fetal bovine serum (FBS) under conditions of 37 °C and 5% CO_2_ in a humidified incubator. Once the HaCaT cells had reached confluence, they were treated with 50 μmol/L DEHP (Sigma-Aldrich) for 72 h; untreated HaCaT cells served as the normal control group (NC group), and A-375 cells served as the melanoma model group (SKCM group). Total cellular RNA was extracted using TRIzol reagent, and cDNA was synthesized via reverse transcription in a 20 μL reaction system using the All-in-One RT Master Mix kit. The synthesized cDNA was diluted 20-fold with RNase-free water, and real-time quantitative PCR (qRT-PCR) was performed using the Q3 SYBR qPCR Master Mix kit in a reaction system containing 1 μL of the diluted cDNA template. PCR amplification was then performed, and the final gene expression levels were calculated using the 2^−ΔΔCt^ method, with GAPDH as the internal reference gene. Three replicates were set up for each reaction to ensure experimental reproducibility. The primer sequences used for qRT-PCR are detailed in Table 1.

**TABLE 1:** Primer sequences of hub genes.

Prime name	Sequence
H-STAT3-F	CGGAGAAGCATCGTGAGTGA
H -STAT3-R	TGCTGCAACTCCTCCAGTTT
H-MYC-F	TCATAACGCGCTCTCCAAGT
H-MYC-R	CGTTCAGAGCGTGGGATGTT
H-HIF1A-F	GCAGCAACGACACAGAAACT
H-HIF1A-R	TGCAGGGTCAGCACTACTTC
H-GAPDH-F	TCCAAAATCAAGTGGGGCGA
H-GAPDH-R	AAATGAGCCCCAGCCTTCTC
H-CTNNB1-F	GCTGGGACCTTGCATAACCT
H-CTNNB1-R	CCAAGCATTTTCACCAGGGC
H -CASP3-F	AGATGGTTTGAGCCTGAGCA
H -CASP3-R	GTGCGTATGGAGAAATGGGC
H-BCL2-F	TCTTTGAGTTCGGTGGGGTC
H-BCL2-R	TCCACAGGGCGATGTTGTC
H-AKT1-F	GGACAAGGACGGGCACATTA
H-AKT1-R	CGACCGCACATCATCTCGTA
H-mTOR-F	GAACCTCCTCCCCTCCAATG
H-mTOR-R	AATTCCACGTACTCAGCGGT
H-IL-6-F	TGAGGAGACTTGCCTGGTGAA
H-IL-6-R	CAGCTCTGGCTTGTTCCTCAC
H-ESR1-F	CCAGATGGTCAGTGCCTTGT
H-ESR1-R	TGCCAGGTTGGTCAGTAAGC
H-CCND1-F	GAGGCGGAGGAGAACAAACA
H-CCND1-R	GGAGGGCGGATTGGAAATGA

### 2.11 Statistical analysis

All data processing and statistical evaluations were conducted utilizing R software, and a p-value of less than 0.05 was considered statistically significant.

## 3 Results

### 3.1 The toxicologic effects of DEHP on melanoma skin cancer

**FIGURE 1:**
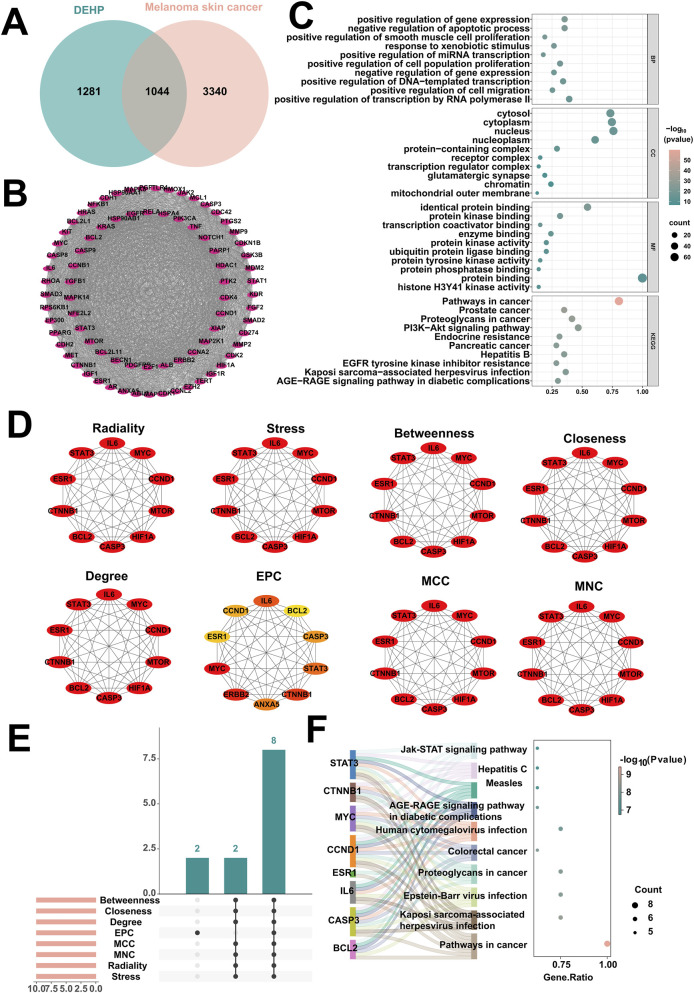
Network toxicology analysis of DEHP promoting melanoma skin cancer. **(A)** Intersection analysis of melanoma skin cancer and DEHP target genes. **(B)** Significant subnetworks in melanoma skin cancer identified by the MCODE plugin. **(C)** Enrichment analysis of the core subnetwork for DEHP-promoted melanoma skin cancer. **(D)** Identification of hub genes by extracting the top 10 genes from the melanoma skin cancer significant subnetwork using eight independent topological algorithms via the CytoHubba plugin. **(E)** The top 10 targets from eight independent topological algorithms jointly determine the core targets of DEHP promoting melanoma skin cancer. **(F)** Enrichment analysis of core targets of melanoma skin cancer.

To elucidate the molecular mechanisms by which DEHP exposure promotes the development of melanoma skin cancer, this study systematically screened key targets and signaling pathways using a combination of network toxicology and bioinformatics methods. First, by intersecting 2,325 potential DEHP targets predicted by STITCH, ChEMBL, Swiss Target Prediction, and PharmMapper with 4,384 melanoma skin cancer targets retrieved from OMIM and GeneCards, a total of 1,044 potential interaction targets were identified (Figure 1A). To further identify core regulatory genes, a progressive PPI network analysis was performed on these 1044 intersecting targets. Initially, the CytoNCA plugin calculated Betweenness, Closeness, and Degree metrics for nodes in the PPI network. Using their respective median thresholds (Betweenness ≥287.18, Closeness ≥0.46, Degree ≥40.5) to identify 418 key genes (Supplementary Table S1). Subsequently, the MCODE plugin performed module mining on this key gene set, identifying a significant subnetwork comprising 79 nodes and 2,558 interactions (Figure 1B). To explore the potential biological functions of this subnetwork, enrichment analysis was performed on the 79 core targets using the DAVID database. This identified 739 GO terms (543 BPs, 75 CCs, 121 MFs) and 150 KEGG pathways, with the top 10 most enriched entries visualized (Figure 1C). Subsequently, to identify hub genes within the network, the CytoHubba plugin was employed to integrate eight topological algorithms: MNC, Degree, EPC, Closeness, Radiality, Betweenness, Stress, and Maximum Subgraph Centrality (MSS). The top 10 potential hub genes were selected for each algorithm (Figure 1D). By integrating these eight sets of results, eight core genes were ultimately identified as being promoted by DEHP exposure in melanoma skin cancer (Figure 1E): BCL2, CASP3, IL6, ESR1, CCND1, MYC, CTNNB1, and STAT3. Finally, KEGG pathway enrichment analysis of these eight core genes (Figure 1F) revealed that the Jak-STAT signaling pathway serves as a key driver of malignant proliferation in melanoma cells. DEHP disrupts this pathway by regulating STAT3, thereby enhancing tumor cell proliferation. These findings elucidate the toxicological molecular mechanism by which DEHP exposure drives melanoma skin cancer progression.

### 3.2 The toxicologic effects of DEHP on squamous cell cancer

**FIGURE 2:**
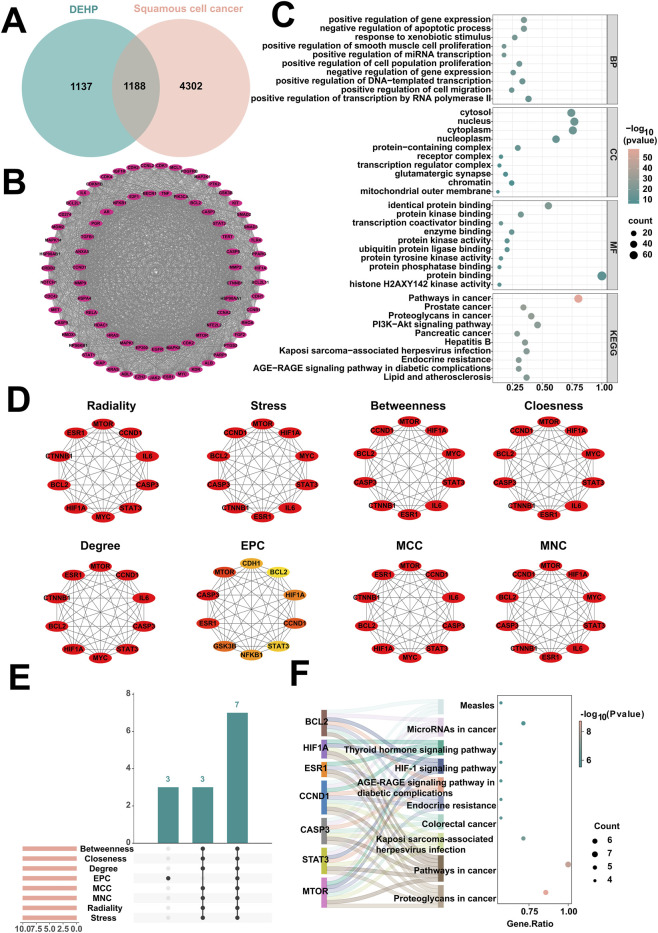
Network toxicology analysis of DEHP promoting squamous cell cancer. **(A)** Intersection analysis of squamous cell cancer and DEHP target genes. **(B)** Significant subnetworks in squamous cell cancer identified by the MCODE plugin. **(C)** Enrichment analysis of the core subnetwork for DEHP-promoted squamous cell cancer. **(D)** Identification of hub genes by extracting the top 10 genes from the squamous cell cancer significant subnetwork using eight independent topological algorithms via the CytoHubba plugin. **(E)** The top 10 targets from eight independent topological algorithms jointly determine the core targets of DEHP promoting squamous cell cancer. **(F)** Enrichment analysis of core targets of squamous cell cancer.

To uncover the molecular mechanisms by which DEHP exposure promotes squamous cell cancer development, this study systematically screen its key target genes and associated pathways. First, through intersection analysis, the study combined 2,325 potential DEHP targets predicted across databases with 5,490 squamous cell carcinoma targets obtained, ultimately identifying 1,188 shared targets (Figure 2A). To identify core regulatory genes among these shared targets, a progressive protein interaction network analysis was conducted. Initially, the CytoNCA plugin calculated Betweenness, Closeness, and Degree metrics for network nodes. Using their respective medians (Betweenness ≥382.96, Closeness ≥0.45, Degree ≥42) as thresholds to identify 460 key genes (Supplementary Table S2). Subsequently, the MCODE plugin performed module mining on this key gene set, identifying a significant subnetwork comprising 78 nodes and 2491 interactions (Figure 2B). To explore the potential biological functions of this subnetwork, enrichment analysis was performed on its 78 core targets using the DAVID database, yielding 731 GO terms (537 BPs, 74 CCs, 120 MFs) and 150 KEGG pathways. The top 10 most enriched entries are visualized in a chart (Figure 2C). Subsequently, to identify hub genes within the network, the CytoHubba plugin was employed. The top 10 potential hub genes were screened for each algorithm (Figure 2D). By integrating the eight sets of screening results, seven core genes promoting squamous cell carcinoma under DEHP exposure were ultimately identified (Figure 2E): MTOR, STAT3, CASP3, CCND1, ESR1, HIF1A, and BCL2. Finally, KEGG pathway enrichment analysis of these seven core genes (Figure 2F) revealed that the HIF-1 signaling pathway is a key regulator of hypoxic adaptation and angiogenesis in squamous cell carcinoma. DEHP disrupts this pathway by modulating HIF1A, thereby enhancing hypoxic tolerance and invasive capacity in squamous carcinoma cells. These findings elucidate the toxicological molecular mechanisms by which DEHP exposure drives squamous cell carcinoma progression.

### 3.3 The toxicologic effects of DEHP on basal cell carcinoma

**FIGURE 3:**
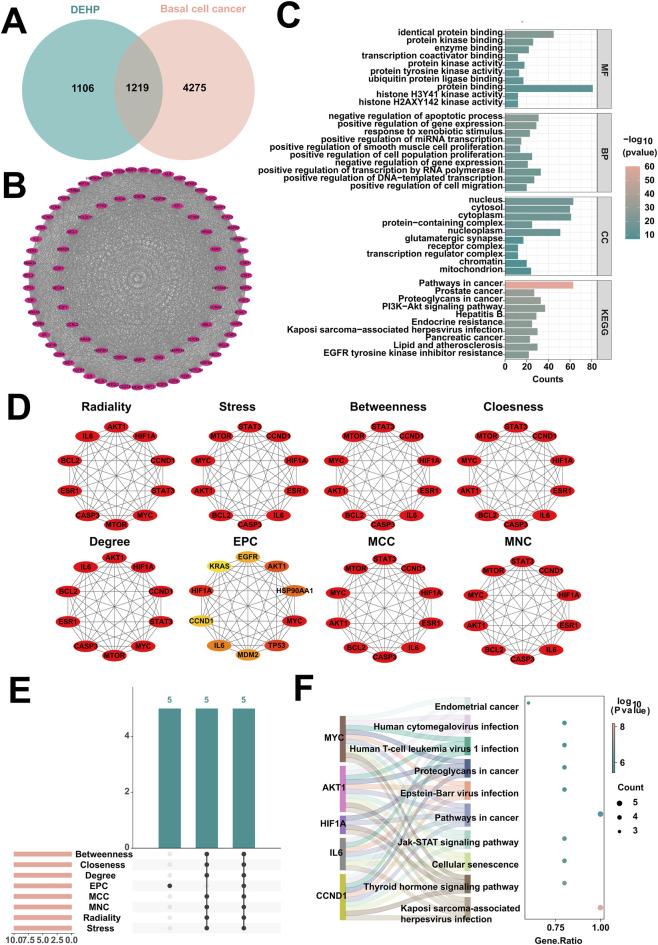
Network toxicology analysis of DEHP promoting basal cell cancer. **(A)** Intersection analysis of basal cell cancer and DEHP target genes. **(B)** Significant subnetworks in basal cell cancer identified by the MCODE plugin. **(C)** Enrichment analysis of the core subnetwork for DEHP-promoted basal cell cancer. **(D)** Identification of hub genes by extracting the top 10 genes from the basal cell cancer significant subnetwork using eight independent topological algorithms via the CytoHubba plugin. **(E)** The top 10 targets from eight independent topological algorithms jointly determine the core targets of DEHP promoting basal cell cancer. **(F)** Enrichment analysis of core targets of basal cell cancer.

To study the molecular mechanisms by which DEHP exposure promotes basal cell carcinoma development, this study systematically screened its key target molecules and associated pathways using network toxicology and bioinformatics approaches. First, an intersection analysis was performed between 2,325 potential DEHP targets, and 5,494 basal cell cancer targets, yielding 1,219 common targets (Figure 3A). To identify core regulatory genes among these shared targets, a progressive protein interaction network analysis was performed. Initially, the CytoNCA plugin calculated Betweenness, Closeness, and Degree metrics for network nodes. Using their respective median thresholds (Betweenness ≥426.06, Closeness ≥0.44, Degree ≥42) as thresholds, identifying 455 key genes (Supplementary Table S3). Subsequently, the MCODE plugin performed module mining on this key gene set, identifying a significant subnetwork comprising 81 nodes and 2,714 interactions (Figure 3B). To explore the potential biological functions of this subnetwork, enrichment analysis was performed on its 81 core targets using the DAVID database. This yielded 804 GO terms (594 BPs, 78 CCs, 132 MFs) and 150 KEGG pathways. The top 10 most enriched entries were visualized (Figure 3C). Subsequently, to identify hub genes within the network, the study employed the CytoHubba plugin to comprehensively apply eight topological algorithms. The top 10 potential hub genes were screened under each algorithm (Figure 3D). By integrating the eight sets of screening results, five core genes were ultimately identified as being promoted by DEHP exposure in basal cell carcinoma (Figure 3E): CCND1, IL6, HIF1A, AKT1, and MYC. Finally, KEGG pathway enrichment analysis of these five core genes revealed that the Jak-STAT signaling pathway is a key regulator of the inflammatory microenvironment and cell proliferation in basal cell carcinoma. DEHP disrupts this pathway by modulating IL6, thereby enhancing the inflammatory response and malignant proliferation capacity of the tumor tissue (Figure 3F). These findings elucidate the toxicological molecular mechanisms by which DEHP exposure drives basal cell carcinoma progression.

### 3.4 Comprehensive toxicological analysis and molecular docking of DEHP in skin cancer

**FIGURE 4:**
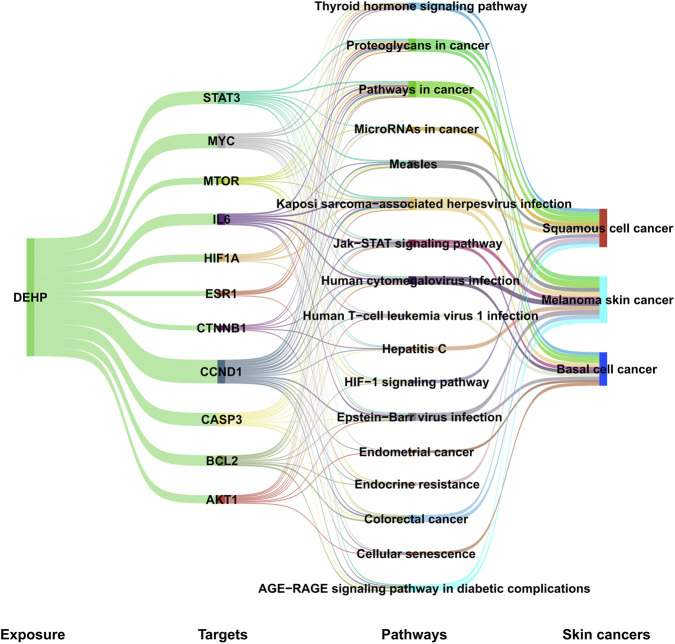
Construction of a comprehensive molecular regulatory network of DEHP in skin cancers, “DEHP-Targets-Pathways-Skin cancers.”

To systematically elucidate the holistic toxicological mechanism by which DEHP exposure drives skin cancer initiation and progression, this study integrated analytical results from the aforementioned skin cancer subtypes to construct a four-tier regulatory network: “DEHP-core target-enriched pathway-skin cancer.” As shown in Figure 4, this network clearly depicts the molecular signaling pathways through which DEHP, as an exogenous disruptor, synergistically promotes the development of melanoma skin cancer, squamous cell cancer, and basal cell cancer by binding to core targets and regulating both common and subtype-specific pathways. This is achieved based on core common targets and integrated pathway information. This network establishes a systematic integration from single subtype analysis to the overall toxicity mechanism of skin cancer. It reveals the core common regulatory axis by which DEHP promotes skin cancer while elucidating the toxicological characteristics of different skin cancer subtypes, providing a visual, multi-level molecular framework for comprehensively understanding DEHP’s skin carcinogenic effects.

**FIGURE 5:**
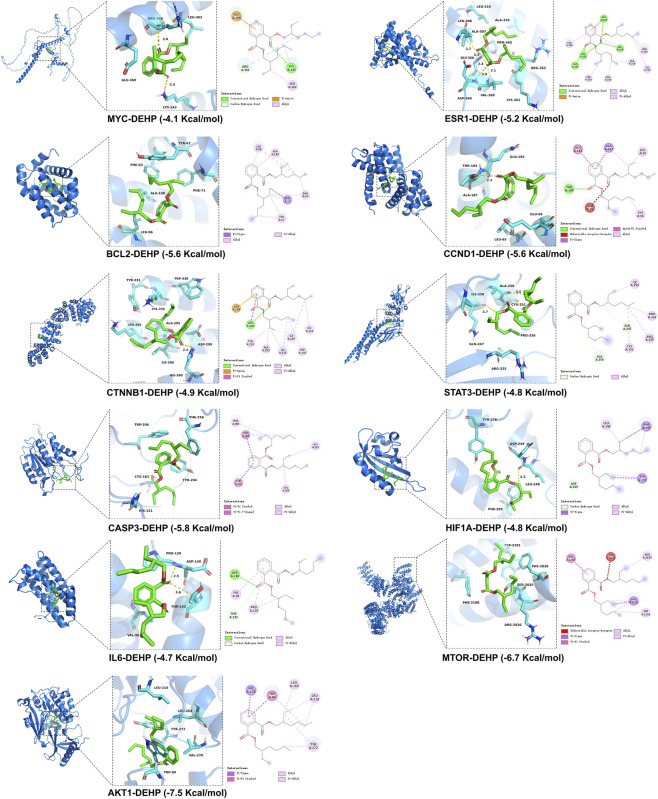
The Structure-based molecular docking of DEHP and key targets (CTNNB1, ESR1, HIF1A, IL6, MTOR, MYC, STAT3, AKT1, BCL2, CASP3, CCND1).

To validate the interaction between core targets and DEHP within this regulatory network, thereby supporting the proposed toxicity mechanism at the molecular level, molecular docking simulations were performed for 11 core target proteins with DEHP. Results demonstrated that DEHP formed stable binding conformations with all target proteins. It embedded into the active pockets of each protein through key intermolecular forces such as hydrogen bonds and hydrophobic interactions, exhibiting high binding affinity (Figure 5). In addition, to ensure the specificity of the docking results, this study selected octocrylene—a compound with similar physicochemical properties but a different structure than DEHP—as a negative control. The docking results showed that octocrylene binds to the core target with lower binding energy than DEHP (Supplementary Figure S1). These findings directly confirm DEHP’s specific binding capacity to core skin cancer targets. This not only validates the reliability of the four-tier regulatory network but also provides crucial molecular evidence for DEHP’s toxic mechanism—directly acting on key targets to regulate downstream signaling pathways and thereby driving skin cancer progression. This lays the foundation for subsequent in-depth mechanistic experiments and the screening of potential intervention targets.

### 3.5 Expression validation of key targets

**FIGURE 6:**
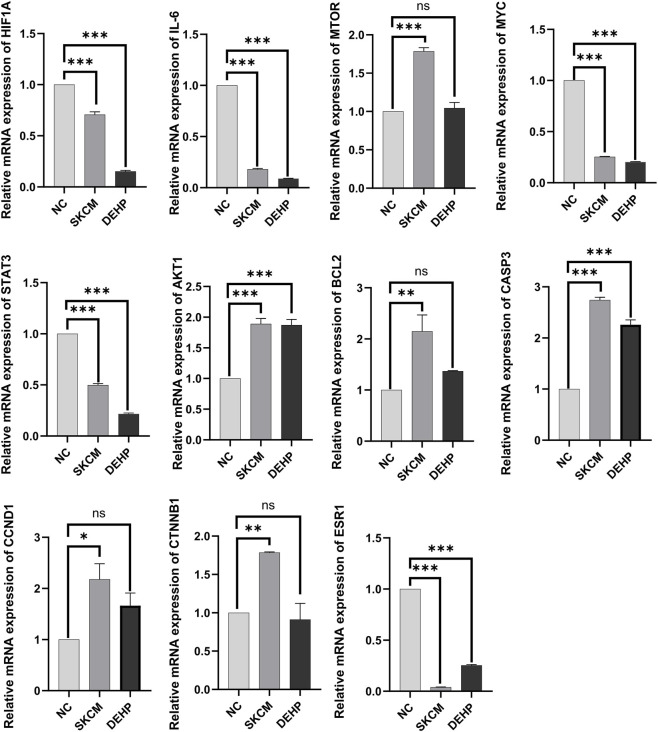
Validation of key targets of SKCM through RT-qPCR. (*p < 0.05, **p < 0.01, ***p < 0.001).

**FIGURE 7:**
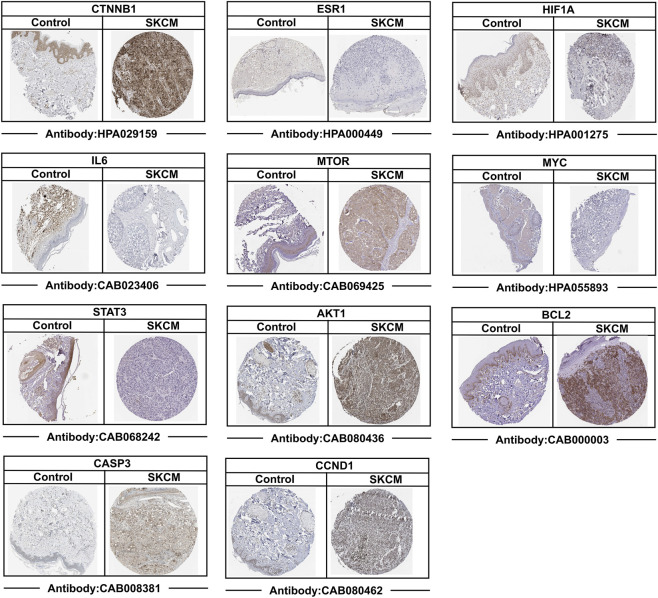
Immunohistochemical (IHC) staining results of key targets (CTNNB1, ESR1, HIF1A, IL6, MTOR, MYC, STAT3, AKT1, BCL2, CASP3, CCND1) in SKCM tissues.

To further validate the abnormal expression of 11 core targets (CTNNB1, ESR1, HIF1A, IL6, MTOR, MYC, STAT3, AKT1, BCL2, CASP3, and CCND1) in skin cancer, this study selected malignant melanoma as a representative model for multi-level validation. Melanoma exhibits the highest malignancy, strong invasive and metastatic capabilities, and significant clinical harm among skin cancers. First, at the transcriptomic level, this study analyzed differential expression based on standardized TCGA tumor samples and GTEx normal tissue RNA sequencing data from the UCSC XENA database, following log2 (TPM+1) transformation. Results revealed significant dysregulation of all 11 core targets in melanoma tissues (Supplementary Figure S2). To experimentally validate the bioinformatics findings and assess the impact of DEHP exposure, we conducted *in vitro* assays. The human malignant melanoma cell line A-375 served as the tumor model, while human immortalized keratinocytes (HaCaT) were used as the normal control. To investigate the effect of DEHP, HaCaT cells were exposed to DEHP. Total RNA was extracted from A-375, untreated HaCaT, and DEHP-treated HaCaT cells, and the mRNA expression levels of the 11 core targets were quantified via RT-qPCR. As shown in Figure 6, the expression trends of these targets in A-375 cells were consistent with the transcriptomic analysis findings, confirming their dysregulation in melanoma. In addition, exposure of normal HaCaT cells to DEHP induced significant expression changes in these same genes. Importantly, the direction of these changes mirrored the aberrant expression patterns observed in A-375 tumor cells. These results demonstrate that DEHP treatment can induce a shift in the expression of key genes in normal cells toward the abnormal pattern observed in melanoma cells. Furthermore, to validate their expression patterns at the protein level, immunohistochemical results based on the Human Protein Atlas (HPA) database were used to compare protein expression of core targets in melanoma tissue versus corresponding normal tissue. Analysis confirmed that the transcriptional abnormalities observed were also present at the protein level (Figure 7). Collectively, these findings reveal abnormal expression patterns of core targets in melanoma at both the transcriptomic and proteomic levels, providing robust experimental evidence that DEHP drives skin cancer initiation and progression by regulating these targets.

## 4 Discussion

Skin cancer is a group of malignant proliferative diseases occurring in the skin, primarily classified pathologically into melanoma and non-melanoma skin cancer (NMSC). Within NMSC, basal cell carcinoma (BCC) and squamous cell carcinoma (SCC) are the most prevalent subtypes (St Denis et al., 2025; Xu et al., 2024). From a pathogenesis perspective, melanoma is typically associated with abnormal cellular proliferation driven by mutations in key genes such as BRAF and NRAS; NMSC frequently involves TP53 gene mutations and chronic inflammatory disturbances within the tumor microenvironment (Ray et al., 2024; Jing et al., 2025). The pathogenesis of skin cancer is influenced by both genetic predisposition and environmental exposure. Beyond ultraviolet radiation as a well-established environmental risk factor, exposure to various environmental pollutants has also been confirmed as a significant pathogenic trigger (Benjamin et al., 2017). Di (2-ethylhexyl) phthalate (DEHP), the most widely used phthalate plasticizer, due to its lack of stable covalent bonds with the polymer matrix, it is prone to leaching from plastic products and migrating into the environment media (air, water, soil, etc.) under unstable environmental conditions such as elevated temperatures, pH fluctuations, or light exposure (Viljoen et al., 2023; Xian et al., 2025). Human exposure to DEHP occurs through multiple pathways including skin contact, dietary intake, and inhalation, leading to its accumulation in body tissues (Eales et al., 2022). Existing epidemiological studies and toxicological experiments indicate an association between DEHP exposure and increased skin cancer risk (Benjamin et al., 2017; Zaman et al., 2025). However, the molecular mechanisms by which DEHP—a widely prevalent environmental endocrine disruptor—contributes to skin cancer initiation and progression remain incompletely elucidated.

Network toxicology is a technology integrating systems biology, bioinformatics, and classical toxicology methods, which systematically deciphers the potential multi-target synergistic toxicity mechanisms of exogenous chemicals (including environmental pollutants, pharmaceuticals, agricultural chemicals, food additives, etc.) by constructing multidimensional interaction networks linking “compounds-targets-pathways-diseases,” providing a panoramic perspective for understanding complex toxicity pathways. In recent years, this approach has been extensively applied to explore the toxic mechanisms of environmental pollutants. For instance, when investigating the association between DEHP and breast cancer, network toxicology identified pivotal genes like HSP90AA1, confirming its multi-pathway synergistic carcinogenic effects (Wang and Wang, 2025). In analyzing DEHP/BPA-induced polycystic ovary syndrome (PCOS), network toxicology identified 26 DEHP-related core targets involving steroid hormone synthesis and apoptosis regulation pathways (Li et al., 2025). In studying the relationship between DEHP and colorectal cancer, network toxicology combined with machine learning screened core targets such as CASP3 and PPARA, revealing its carcinogenic promotion through reshaping the intestinal immune microenvironment (Wang et al., 2025a). In investigating DEHP-induced non-small cell lung cancer (NSCLC), network toxicology identified 225 overlapping targets, confirming the critical roles of the PI3K-Akt pathway and TP53, AKT1 (Chen et al., 2025). In analyzing DEHP-induced prostate cancer, network toxicology identified 88 associated targets, confirming that SRC, MMP9, and others mediate toxicity through the focal adhesion pathway (Chen et al., 2025). These studies systematically elucidated DEHP’s multi-target toxicological characteristics through network toxicology techniques, establishing a methodological foundation for elucidating the mechanism by which DEHP induces skin cancer in this research.

The aforementioned studies demonstrate that network toxicology can multidimensionally elucidate the toxicological mechanisms linking environmental exposures to disease. Therefore, this study systematically elucidates the toxicological mechanism by which DEHP exposure promotes skin cancer through the integration of network toxicology, molecular docking, and experimental validation. Specifically, during the target screening phase, this study combined databases such as STITCH and ChEMBL to predict potential DEHP targets. Targets associated with skin cancers (melanoma, squamous cell carcinoma, basal cell carcinoma) were obtained from OMIM and GeneCards databases. Shared targets were identified through intersection analysis. Subsequently, a protein-protein interaction (PPI) network was constructed using STRING. Through CytoNCA topological analysis, MCODE module identification, and CytoHubba algorithm integration, 11 key targets were ultimately identified: CTNNB1, ESR1, HIF1A, IL6, MTOR, MYC, STAT3, AKT1, BCL2, CASP3, and CCND1. At the mechanism level, GO/KEGG enrichment analysis revealed these targets are involved in cell proliferation, apoptosis, inflammatory responses, and core pathways including Jak-STAT, HIF-1, and PI3K-Akt/mTOR, exhibiting subtype-specific regulatory patterns across different skin cancer subtypes. At the molecular validation level, molecular docking results confirmed that DEHP forms stable binding conformations with all 11 key targets, embedding into active pockets via hydrogen bonds and hydrophobic interactions, with binding energies meeting criteria for stable interactions. Experimental validation using TCGA/GTEx transcriptomic data, HPA proteomic data, and A-375/Hacat cell RT-qPCR experiments confirmed significant dysregulation of these 11 targets in melanoma tissues and tumor cells. Collectively, these findings not only establish the central regulatory role of these 11 key targets in DEHP-induced skin carcinogenesis but also construct a regulatory chain—“DEHP → core targets → pathways → skin cancer”—through subtype-specific pathway analysis and multi-level validation. This provides comprehensive and robust experimental and computational evidence for the skin carcinogenic mechanism of DEHP.

Notably, the key DEHP-induced skin cancer targets identified through network toxicology in this study have been previously demonstrated to play crucial regulatory roles in dermatological diseases. For instance, STAT3 participates in skin cancer development through abnormal activation, with elevated phosphorylation levels promoting skin cancer cell proliferation and inhibiting apoptosis (Park et al., 2022). ESR1 functions as a pivotal node in OLFM4-induced signaling pathways, participating in psoriasis pathogenesis by regulating keratinocyte proliferation and migration (Klaas et al., 2022). HIF1A modulates the MAPK pathway to influence the pathogenesis of methicillin-resistant *Staphylococcus aureus* (MRSA)-mediated skin infections, with its expression changes affecting lesion size and inflammatory response intensity (Yadav et al., 2024). BCL2 exhibits differential expression in non-melanoma skin cancers, particularly basal cell carcinoma, with significantly higher expression levels in non-invasive tumors compared to invasive ones (Prídavková et al., 2024). As a key molecule in the PI3K/Akt/mTOR pathway, abnormal activation of mTOR contributes to DMBA/TPA-induced skin carcinogenesis (Wang Z. et al., 2025). AKT1, a key molecule in the PI3K/AKT pathway, is frequently downregulated in cutaneous squamous cell carcinoma and contributes to tumor pathogenesis (Ha et al., 2010). MYC can be activated by the secretome of cutaneous squamous cell carcinoma (cSCC) cells. Through the mTOR/MYC pathway, it promotes keratinocytes to acquire a tumor-like phenotype characterized by enhanced proliferation and migration, thereby participating in cSCC development (Hoesl et al., 2020). The above studies collectively confirm that the key targets identified in this research are closely associated with the pathological processes and cellular functional regulation of skin diseases, fully demonstrating the scientific validity and reliability of the target screening approach employed in this study.

Furthermore, molecular docking, as a key technology in computational toxicology and structural biology, provides structural biology-level prediction and validation for molecular recognition between compounds and targets by simulating the three-dimensional spatial conformation matching between small-molecule compounds and target proteins (Hasan et al., 2025; Huang et al., 2025). In this study, molecular docking plays a crucial role in elucidating the pathogenic mechanism linking DEHP to skin cancer. Specifically, molecular docking validates the binding potential between DEHP and core targets identified through network toxicology screening—including STAT3, IL-6, ESR1, and HIF1A—by assessing interaction stability through binding energy values. This provides direct molecular evidence for the regulatory network linking “DEHP-target-pathway-skin cancer.” Second, molecular docking precisely reveals specific binding sites and interaction patterns between DEHP and target proteins, providing a structural foundation for elucidating how DEHP modulates target functions. It clarifies whether DEHP affects normal biological functions by occupying active sites or disrupting target conformations (Park et al., 2022). In summary, molecular docking serves as a crucial technical tool in network toxicology. It not only enhances the scientific rigor and reliability of core target screening in this study but also provides essential structural biology support for systematically elucidating the molecular mechanisms of DEHP-induced skin carcinogenesis.

Although this study elucidated the core target and potential mechanism of DEHP-induced skin cancer through network toxicology combined with molecular docking systems, several limitations warrant acknowledgment. First, molecular docking only simulates the static binding patterns between DEHP and target proteins, failing to account for the complex physiological environment *in vivo*. Consequently, it cannot fully replicate the dynamic regulatory processes occurring within the body. Second, this study constitutes computational predictive research, the conclusions presented are still at an early stage, and future research will need to expand the sample cohort to conduct multi-level validation (such as epidemiological statistical analysis, cellular and animal experiments, etc.), which will be the focus of our future research efforts.

## 5 Conclusion

In summary, this study systematically elucidates the toxicological mechanisms by which DEHP exposure promotes skin cancer through the integration of network toxicology, molecular docking, and experimental validation. Specifically, the study identified 11 key targets—CTNNB1, ESR1, HIF1A, IL6, MTOR, MYC, STAT3, AKT1, BCL2, CASP3, and CCND1—exhibiting subtype-specific regulatory patterns in melanoma, squamous cell carcinoma, and basal cell carcinoma. Concurrently, the study confirmed that DEHP forms stable binding conformations with these 11 key targets, thereby synergistically driving abnormal proliferation of skin cells, suppression of apoptosis, and imbalance in the inflammatory microenvironment. Subsequently, a four-tier regulatory network—“DEHP - Core Targets - Pathways - Skin Cancer”—was constructed, providing a systems biology perspective for understanding the association between DEHP and skin cancer. Furthermore, transcriptomic (TCGA/GTEx), proteomic (HPA), and *in vitro* cellular assays (RT-qPCR) collectively validated significant dysregulation of these targets in skin cancer tissues and tumor cells, providing robust experimental evidence for DEHP’s regulatory role. In summary, this study not only fills a gap in understanding the molecular mechanisms by which DEHP induces skin cancer but also provides novel biomarkers for environmental exposure prevention and targeted interventions against skin cancer.

## Data Availability

The datasets presented in this study can be found in online repositories. The names of the repository/repositories and accession number(s) can be found in the article/Supplementary Material.
