# Supplementary material for: Revealing key biomarkers and molecular mechanisms associated with di(2-ethylhexyl) phthalate in skin cancer
Source: Front Mol Biosci. 2026 May 21;13:1841556. doi: 10.3389/fmolb.2026.1841556 (PMC13233234; doi:10.3389/fmolb.2026.1841556)
Supplement: Supplementary file 1 [file Table2.docx]

**
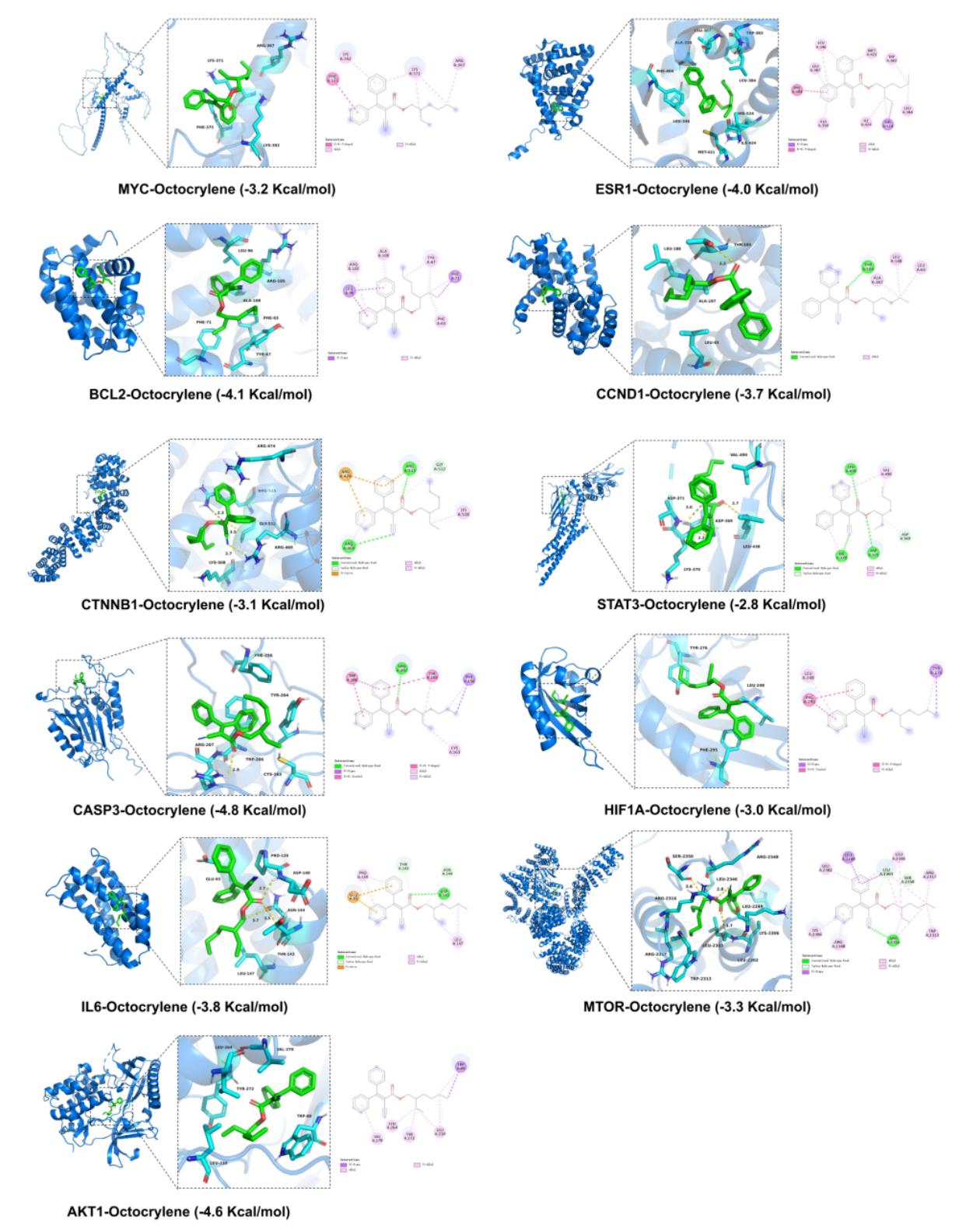
**

**Figure S1.** The Structure-based molecular docking of octocrylene and key targets (CTNNB1, ESR1, HIF1A, IL6, MTOR, MYC, STAT3, AKT1, BCL2, CASP3, CCND1).

**
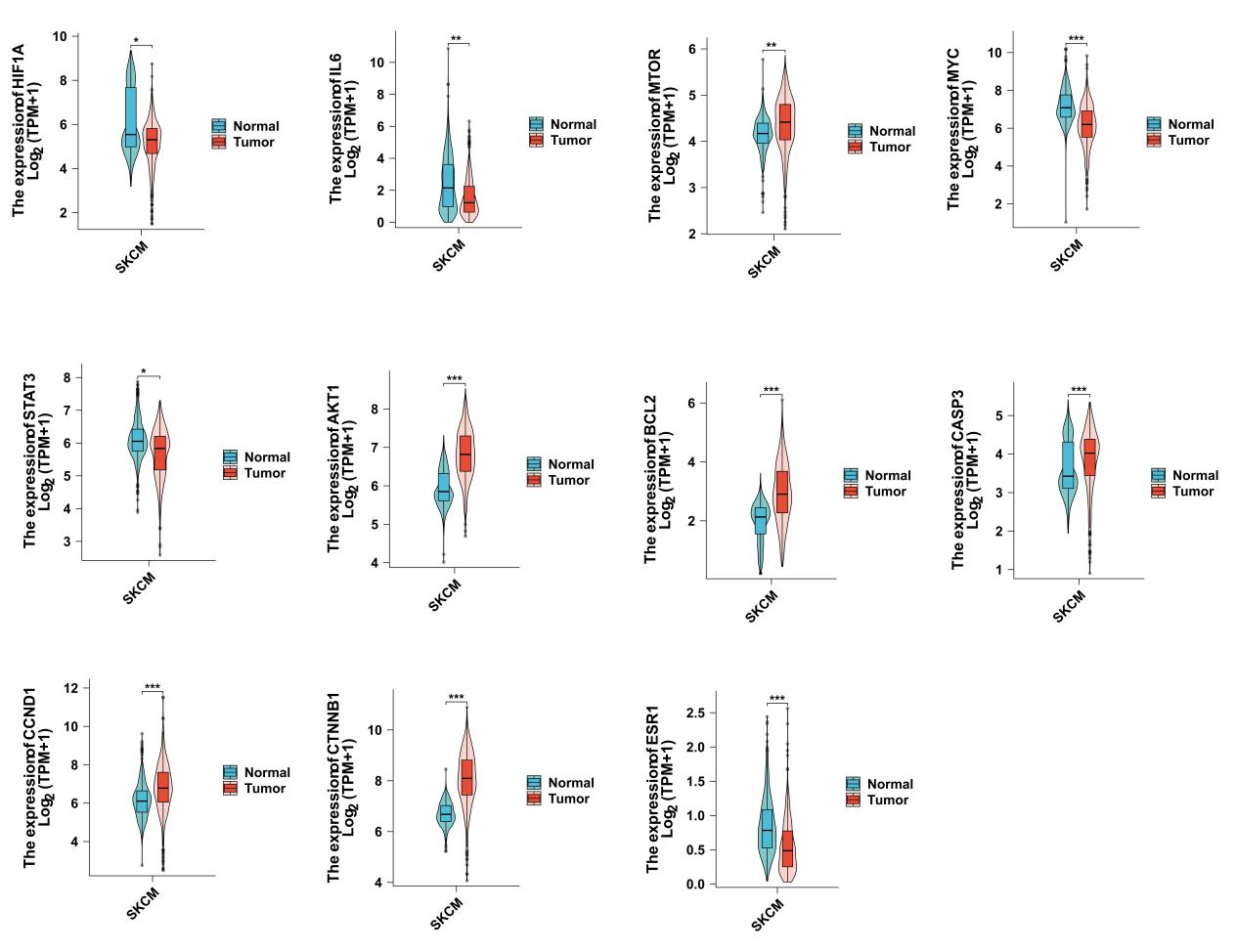
Figure S2.** Differential expression of 11 key targets (CTNNB1, ESR1, HIF1A, IL6, MTOR, MYC, STAT3, AKT1, BCL2, CASP3, CCND1) at the transcriptome level. The data were derived from TCGA tumor samples (SKCM) and corresponding normal tissue samples from GTEx.
